# A Case of Non‐Small‐Cell Lung Cancer With Massive Malignant Ascites Treated With Chemotherapy Combined With Cell‐Free and Concentrated Ascites Reinfusion Therapy

**DOI:** 10.1111/1759-7714.70074

**Published:** 2025-04-24

**Authors:** Koichi Jingo, Haruki Hirakawa, Tomoyasu Mimori, Shinya Fujioka, Yuki Muto, Makiko Komaru, Manami Haba, Yoichiro Mitsuishi, Kazuhisa Takahashi

**Affiliations:** ^1^ Department of Respiratory Medicine Juntendo University Tokyo Japan

**Keywords:** carcinomatous peritonitis, cell‐free and concentrated ascites reinfusion therapy (CART), lung cancer

## Abstract

We report the case of a 65‐year‐old woman with stage IVB lung adenocarcinoma who developed malignant ascites during treatment. Despite multiple ascitic fluid drainages and second‐line chemotherapy, the ascites progressively worsened. The initiation of cell‐free and concentrated ascites reinfusion therapy (CART) led to improved abdominal distention, increased blood albumin levels, and slower ascites accumulation. To our knowledge, this is the first report of CART combined with chemotherapy for the management of malignant ascites associated with lung cancer.

## Graphical abstract

A 65‐year‐old woman with stage IVB lung adenocarcinoma developed progressive malignant ascites despite conventional drainage and chemotherapy. Cell‐free and concentrated ascites reinfusion therapy (CART) was initiated; it relieved symptoms, increased serum albumin levels, and reduced ascites accumulation, allowing prolonged chemotherapy. This is the first reported case of CART combined with chemotherapy for malignant ascites in lung cancer.

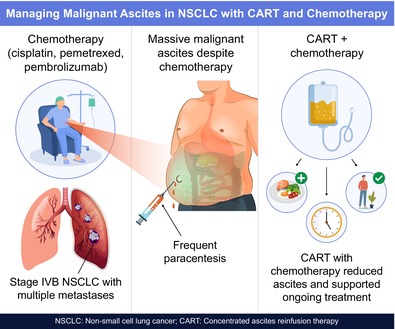

## 1 Introduction

Cell‐free and concentrated ascites reinfusion therapy (CART), introduced in 1971 by Levy for patients with cirrhosis [1], involves filtering ascitic fluid to remove bacteria, cancer cells, and blood components, concentrating albumin and other substances, and reinfusing the fluid intravenously. In Japan, CART has been insurance‐covered since 1981 for refractory ascites, typically once every 2 weeks. A cohort study of 37 patients with malignant ascites, primarily due to gastric cancer, and none with lung cancer demonstrated a significant improvement in life‐obstacle scores [2]. We report a case of massive ascites from carcinomatous peritonitis due to non‐small cell lung cancer (NSCLC), which was controlled with CART, allowing prolonged chemotherapy and a stable condition.

## 2 Case Presentation

A 65‐year‐old woman presented with dyspnea. A Bronchoscopic biopsy of the right upper lobe showed adenocarcinoma. Imaging revealed multiple bone and brain metastases, leading to a diagnosis of lung adenocarcinoma (cT4N2M1c, stage IVB), per the UICC TNM classification (8th edition). No driver gene mutations or rearrangements were detected, but the programmed death‐ligand 1 tumor proportion score was 60%. She received palliative radiation for spinal and brain metastases and started first‐line chemotherapy with cisplatin, pemetrexed, and pembrolizumab.

After 13 chemotherapy cycles, abdominal computed tomography (CT) revealed ascites, confirmed as adenocarcinoma by cytological analysis. Progressive disease (PD) was diagnosed. Docetaxel and ramucirumab (DTX + RAM) were introduced as second‐line therapy. Ascites worsened, necessitating frequent abdominal paracentesis, and activities of daily living (ADL) declined. CART was initiated with patient consent.

**FIGURE 1:**
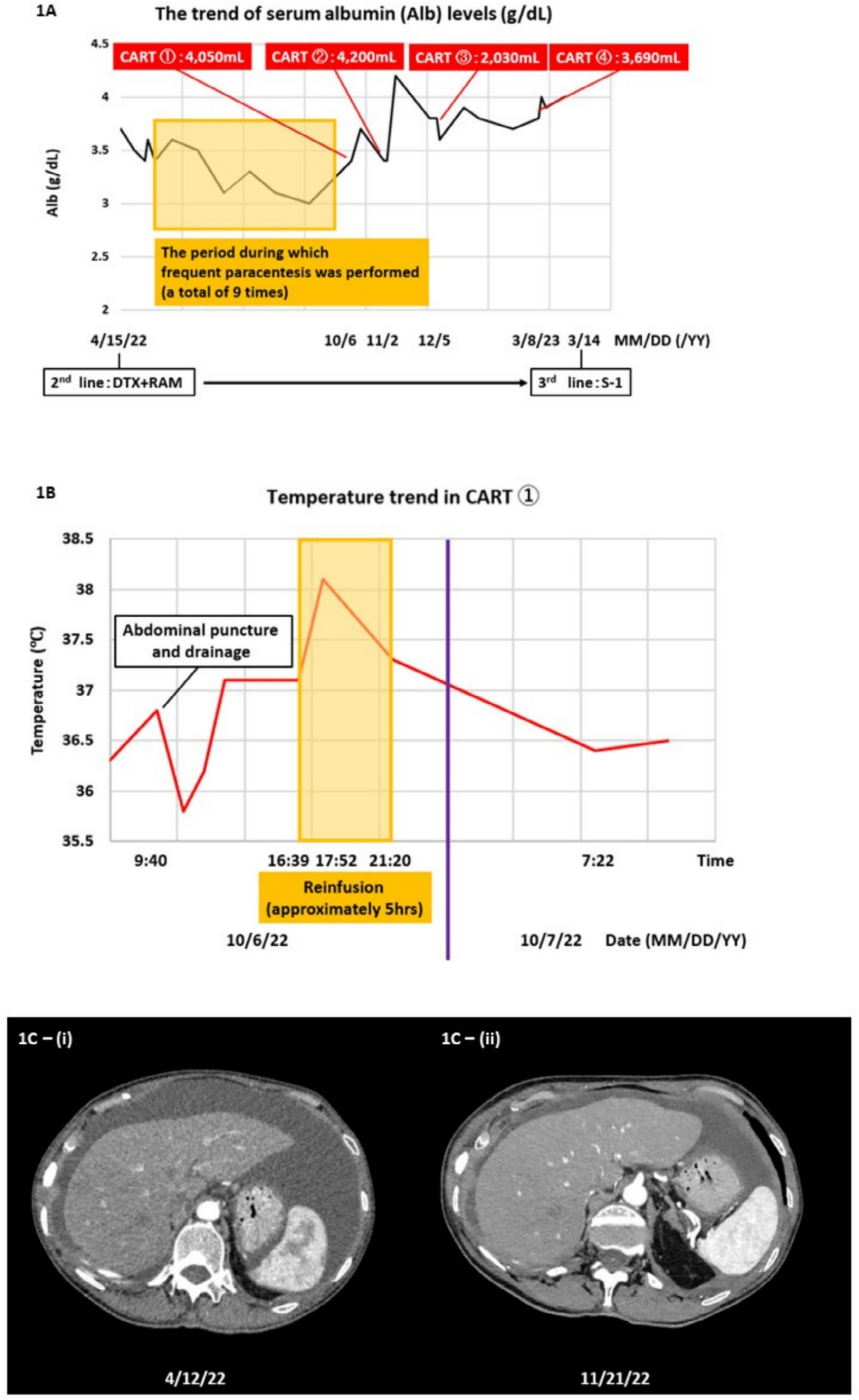
The trend of serum albumin levels. Before the introduction of CART, peritoneal puncture was performed frequently, with nine punctures conducted over approximately 4.5 months. The average interval between punctures was 17.0 days (7–35 days). In contrast, after the introduction of CART, the average interval between CART sessions was 53.0 days (27–89 days), indicating an extension of the puncture intervals. The volume of ascitic fluid drained per puncture ranges from 300 to 2500 mL during peritoneal puncture, whereas with CART, it ranges from 2030 to 4200 mL, allowing a large volume of fluid to be drained in a single session. CART, cell‐free and concentrated ascites reinfusion therapy; DTX, docetaxel; RAM, ramucirumab; S‐1, tegafur‐gimeracil‐oteracil potassium. (B) Temperature during the first CART: As an example of temperature progression during CART, the patient's temperature trend following the first CART procedure is depicted in the graph. Abdominal puncture was performed at 9:40 AM, resulting in 4050 mL of ascitic fluid drainage with a total protein concentration of 5.4 g/dL. Filtration and concentration were then performed, yielding 520 mL of concentrated ascitic fluid with a total protein concentration of 22 g/dL. Reinfusion commenced at 4:39 PM at a rate of approximately 100 mL/h. A fever of 38.1°C was noted 1 h and 13 min after the initiation of reinfusion; however, by the time the reinfusion was completed (approximately 5 h later), the temperature had decreased to 37.3°C. By the following morning, the patient's temperature had returned to baseline, and no further complications were observed. (C) CT findings of ascites with contrast: The cross‐sectional images of the liver on contrast‐enhanced CT are shown. (C‐i) was obtained on April 12, 2022, just prior to the first course of DTX + RAM on April 12, 2022. (C‐ii), obtained on November 21, 2022, shows findings after the second CART (administered on November 2, 2022), with a noticeable decrease in ascites.

**FIGURE 2:**
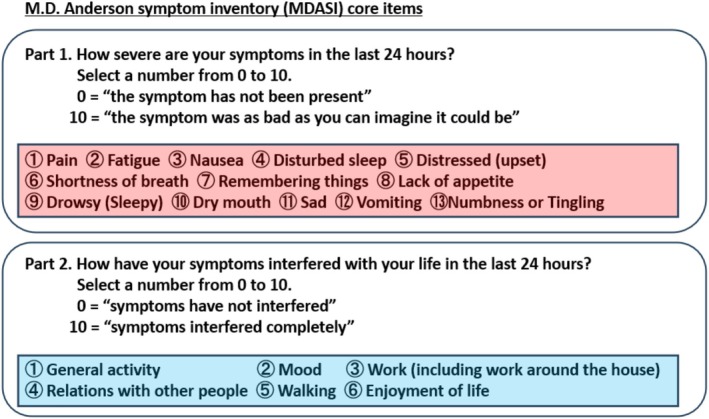
M.D. Anderson symptom inventory (MDASI) core items [3]. Modified based on the study by Cleeland et al. The MDASI is a symptom assessment index in which the severity of each symptom (13 items, highlighted in red squares) and the degree of interference with daily activities (6 items, highlighted in blue squares), totaling 19 items, were self‐assessed on a scale from 0 to 10 within 24 h.

**FIGURE 3:**
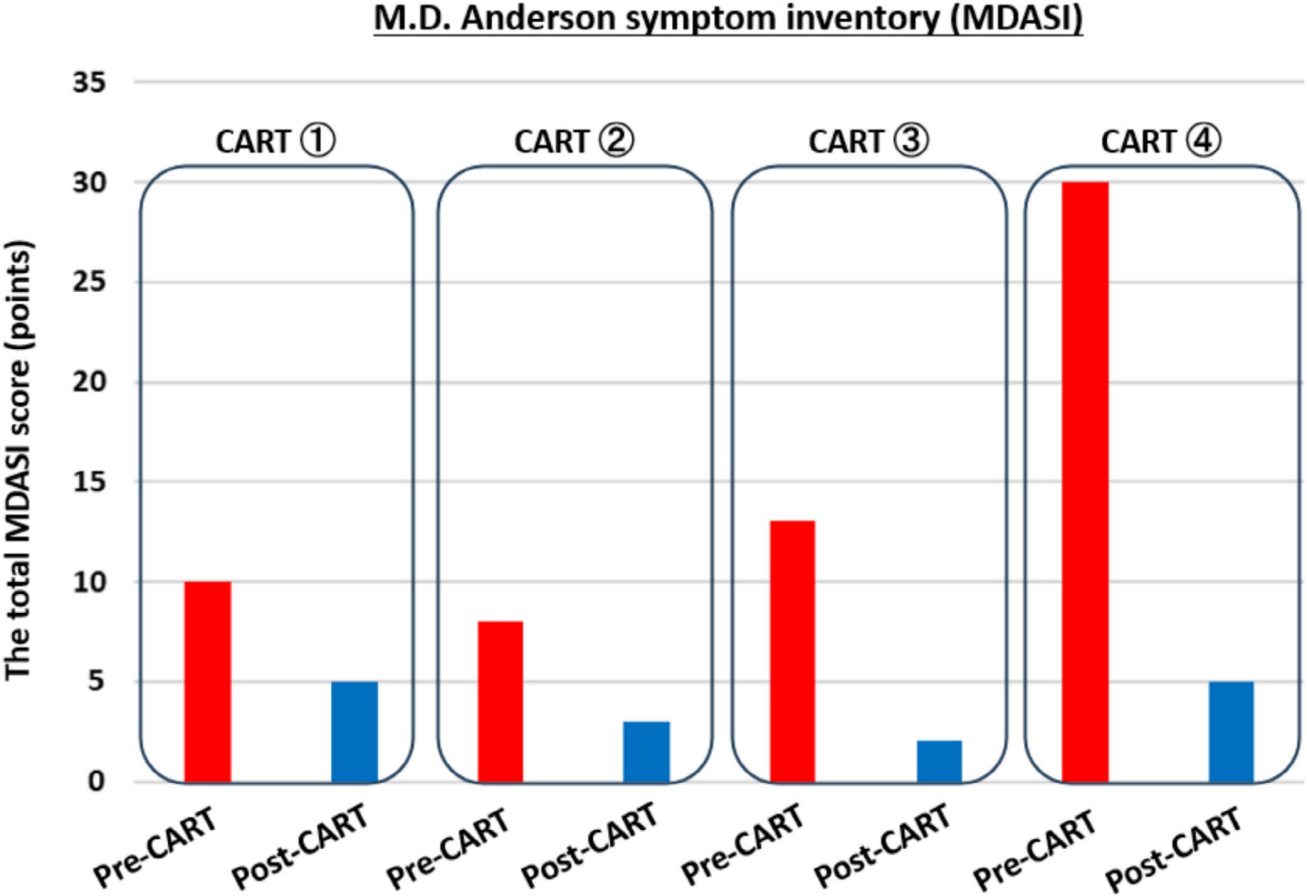
The transition of the total MDASI score in this case. The total MDASI scores for the four CART sessions in this study are summarized. The red bar graph represents the MDASI scores before CART, whereas the blue bar graph represents the scores immediately after CART. There is no consensus regarding the minimally clinically important differences for each item in the MDASI. According to the MDASI User Guide version 1, values ranging from 0.98 to 1.21 have been proposed [4], suggesting meaningful improvement in this case. Notably, the MDASI score before the fourth CART session was elevated due to a delay in hospitalization caused by the patient's extended holiday period. Nevertheless, the MDASI scores consistently improved after each session compared to those before each session.

Upon admission, her performance status (PS) was 1, and her abdomen was markedly distended (91 cm). Blood tests showed hypoalbuminemia (albumin 3.4 g/dL). Before CART, paracentesis averaged 1400 mL of fluid removal per procedure. After CART, the volume of fluid removed per procedure increased to an average of approximately 3500 mL, and paracentesis frequency decreased. Serum albumin improved and stabilized at ≥ 3.5 g/dL (Figure 1A). PS remained stable at 1, with no new metastases. Subjective symptoms were assessed using the M.D. Anderson Symptom Inventory (MDASI) significantly reduced after CART (Figures 2 and 3).

After 11 cycles of DTX + RAM, the disease was evaluated as PD based on worsening ascites and increasing tumor marker levels, leading to a switch to third‐line therapy with tegafur, gimeracil, and oteracil potassium (S‐1). As ADL remained preserved, chemotherapy was continued.

## 3 Discussion

In this case, CART prolonged the puncture interval, improved the patient's nutritional status, and maintained PS, enabling prolonged chemotherapy. Peritonitis carcinomatosa in NSCLC occurs in 0.84% of cases, with a median survival of 2.8 months [5]. The combination of DTX + RAM used in this case is well established, based on the REVEL trial [6], which reported a median overall survival of 10.5 months. In this case, the patient received chemotherapy for approximately 1 year, surpassing the median survival. This prolonged survival is likely attributable to CART's ability to maintain PS.

Reported adverse reactions to CART include fever, chills, shock, and hypertension, with fever being the most common (20.5%). These reactions are typically managed with corticosteroids, NSAIDs, and other supportive treatments [7]. In this case, hydrocortisone (100 mg) and dl‐chlorpheniramine maleate (10 mg) were administered prophylactically, and the reinfusion rate was maintained at 100 mL/h. Consequently, only a mild transient fever was observed (Figure 1B), and CART was safely completed.

No reports have described a combination of CART and chemotherapy for lung cancer within the scope of our search. Several studies have supported the use of CART in combination with chemotherapy for gastrointestinal cancers. A retrospective study of 30 patients with gastric cancer confirmed the safety of combined CART and chemotherapy, with 127 CART procedures performed [8]. Another report of 21 gastric and 9 colorectal cancer cases showed a prolonged interval between abdominal punctures in 83% of gastric cancer cases and 100% of colorectal cancer cases [9]. In this study, PS remained stable in 80% of the patients and improved in 20% after CART. Similarly, no severe adverse events occurred in this case; the puncture intervals were prolonged, and PS showed no deterioration, consistent with previous findings.

In this case, MDASI transitions show CART improved subjective symptoms, allowing chemotherapy to continue for 1 year without PS decline. Consistent with previous reports, this case demonstrates CART's efficacy in improving subjective symptoms and overall condition while reducing abdominal paracentesis frequency. This suggests CART may be a valuable treatment for peritoneal carcinomatosis in patients with lung cancer, particularly when abdominal paracentesis control is inadequate.

The ascites were considered to have transudative and exudative components. CART was presumed to increase ascitic fluid removal per session and exert effects through concentration and reinfusion, improving hypoalbuminemia.

CART concentrates and reinfuses not only albumin but also other proteins, including cytokines with potential antitumor effects and α1‐acid glycoprotein, which may influence the pharmacokinetics of anticancer drugs like docetaxel [10], leading to elevated protein levels in the blood. However, this retrospective case report of chemotherapy combined with CART did not evaluate cytokine or protein concentrations in the blood and ascitic fluid. The primary aim was to demonstrate the clinical efficacy of CART without assessing cost‐effectiveness or accessibility in detail. In addition, the rarity of carcinomatous ascites in lung cancer makes prospective studies challenging. These limitations should be acknowledged. However, with further case accumulation, CART may be considered for integration into standard treatment protocols.

In conclusion, CART was associated with (i) prolonged puncture intervals, (ii) improved nutritional status, and (iii) maintenance of PS. We believe CART provides useful supportive care to maintain general health and sustain chemotherapy.

## Author Contributions

Conceptualization: Haruki Hirakawa and Tomoyasu Mimori. Investigation: Koichi Jingo, Haruki Hirakawa, Tomoyasu Mimori, Shinya Fujioka, Yuki Muto, Makiko Komaru, and Manami Haba. Resources: Koichi Jingo, Haruki Hirakawa, Yuki Muto, Makiko Komaru, and Manami Haba. Writing – original draft: Koichi Jingo. Writing – review and editing: Koichi Jingo, Haruki Hirakawa, Tomoyasu Mimori, Shinya Fujioka, Yuki Muto, Makiko Komaru, Manami Haba, Yoichiro Mitsuishi, and Kazuhisa Takahashi. Supervision: Kazuhisa Takahashi. Visualization: Koichi Jingo.

## Consent

Consent for the publication of this article was obtained from the patient's family.

## Conflicts of Interest

The authors declare no conflicts of interest.

## Data Availability

Data sharing is not applicable to this article as no new data were created or analyzed in this study.
